# The Ubiquitin-Proteasome System Plays Dual Roles in Plant Antiviral Defense and Viral Pathogenicity

**DOI:** 10.3390/plants15172648

**Published:** 2026-08-29

**Authors:** Ziru Chu, Donghai Wang, Wangyun Zhang, Jianping Chen, Fei Yan, Shaofei Rao

**Affiliations:** State Key Laboratory for Quality and Safety of Agro-Products, Key Laboratory of Biotechnology in Plant Protection of MARA, Zhejiang Key Laboratory of Green Plant Protection, Institute of Plant Virology, Ningbo University, Ningbo 315211, China

**Keywords:** ubiquitin-proteasome system, ubiquitination, plant viruses, host–pathogen interactions, immune evasion

## Abstract

The ubiquitin-proteasome system (UPS) constitutes a highly conserved regulatory hub governing protein turnover and signal transduction in eukaryotes, which precisely determines the fate of substrate proteins via dynamic and reversible ubiquitination. During long-term coevolution between plants and viruses, the UPS has evolved into a critical battlefield for host–virus arms races. Plants exploit the substrate recognition specificity and proteolytic activity of the UPS to selectively eliminate essential viral proteins required for infection, thereby establishing multilayered antiviral immune barriers. In contrast, viruses have evolved diverse effector proteins to antagonize or hijack this pathway to facilitate their replication and spread. Competitive exploitation of this shared regulatory machinery underlies the fundamental logic of bidirectional regulation in plant–virus interactions. This review systematically summarizes the molecular basis of UPS-mediated plant antiviral immunity, as well as convergent pathogenic strategies adopted by diverse viruses to perturb ubiquitin signaling, suppress host immune responses, and reprogram the intracellular environment. The work aims to provide theoretical insights for deciphering viral pathogenesis and breeding crops with durable virus resistance.

## 1. Introduction

### 1.1. Plant Viral Diseases

Plant viruses are obligate intracellular pathogens transmitted by insect vectors, mechanical wounding and other routes. Characterized by highly compact genomes encoding a limited set of multifunctional proteins, plant viruses must hijack host organelles, metabolic pathways and protein regulatory networks to complete replication, virion assembly and cell-to-cell movement [1,2,3]. Major staple crops including rice, wheat and maize, as well as cash crops such as cotton, tomato and potato, are persistently threatened by diverse plant viruses, posing severe risks to global agricultural stability. Distinct from fungal and bacterial pathogens, viruses devoid of cellular structures, making chemical control largely ineffective. Accordingly, unraveling endogenous plant immune pathways and dissecting the molecular mechanisms underlying viral immune evasion lays the theoretical foundation for breeding broad-spectrum virus-resistant crops [4].

During viral infection, host defense-related proteins and viral proteins undergo continuous synthesis and dynamic turnover. Compared with transcriptional regulation, post-translational modifications rapidly and reversibly modulate the abundance and biological activity of target proteins, serving as vital mechanisms to rewire intracellular signaling networks. Among all post-translational modifications, ubiquitination targets the broadest spectrum of substrates. The ubiquitin-proteasome system (UPS) operates throughout the activation of host antiviral immunity and viral infection, exerting pivotal functions in plant–virus interactions [5].

### 1.2. Core Components and Biochemical Mechanisms of the UPS

**Figure 1:**
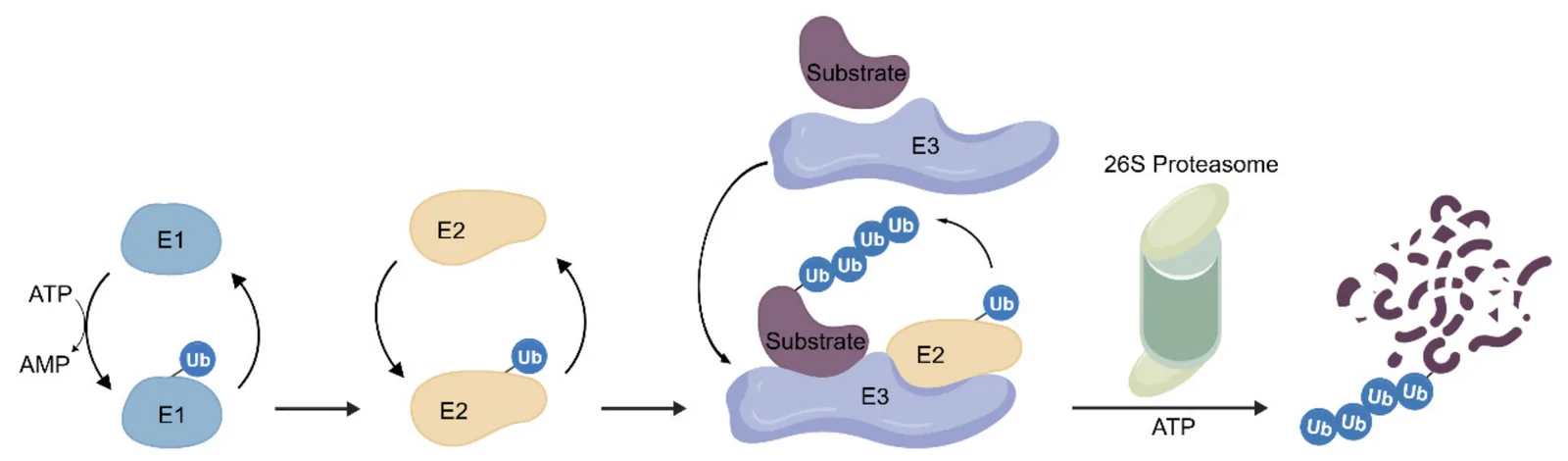
Schematic diagram of the ubiquitin-proteasome cascade. Ubiquitin-activating enzyme (E1) activates free ubiquitin in an ATP-dependent manner. The activated ubiquitin is subsequently transferred to the ubiquitin-conjugating enzyme (E2) via a thioester bond. E2 cooperates with ubiquitin ligase (E3) to covalently attach ubiquitin to lysine residues of substrate proteins. Substrates tagged with polyubiquitin chains are specifically recognized by the 26S proteasome and degraded into peptide fragments. Figure created with BioGDP.com [8].

Ubiquitin (Ub) is a highly conserved 76-amino-acid polypeptide ubiquitous across eukaryotes. The ubiquitin sequences from yeast, humans and plants differ at merely three amino acid residues [6]. Ubiquitination mediated by the UPS is a conserved three-step enzymatic cascade, in which ubiquitin molecules are covalently attached to lysine residues of substrate proteins via sequential reactions catalyzed by E1 ubiquitin-activating enzymes, E2 ubiquitin-conjugating enzymes and E3 ubiquitin ligases: (1) E1 ubiquitin-activating enzyme: Using energy released from ATP hydrolysis, E1 catalyzes formation of a high-energy thioester bond between the C-terminal glycine of ubiquitin and the active-site cysteine of E1 to activate free ubiquitin. (2) E2 ubiquitin-conjugating enzyme: Activated ubiquitin is transferred from E1 to E2 via a transesterification reaction, generating an E2-Ub thioester intermediate. (3) E3 ubiquitin ligase: The primary determinant of substrate specificity within the cascade. E3 simultaneously recruits Ub-charged E2 enzymes and specifically recognizes target substrates, catalyzing formation of an isopeptide bond that links ubiquitin to substrate lysine residues [7] (Figure 1).

**Figure 2:**
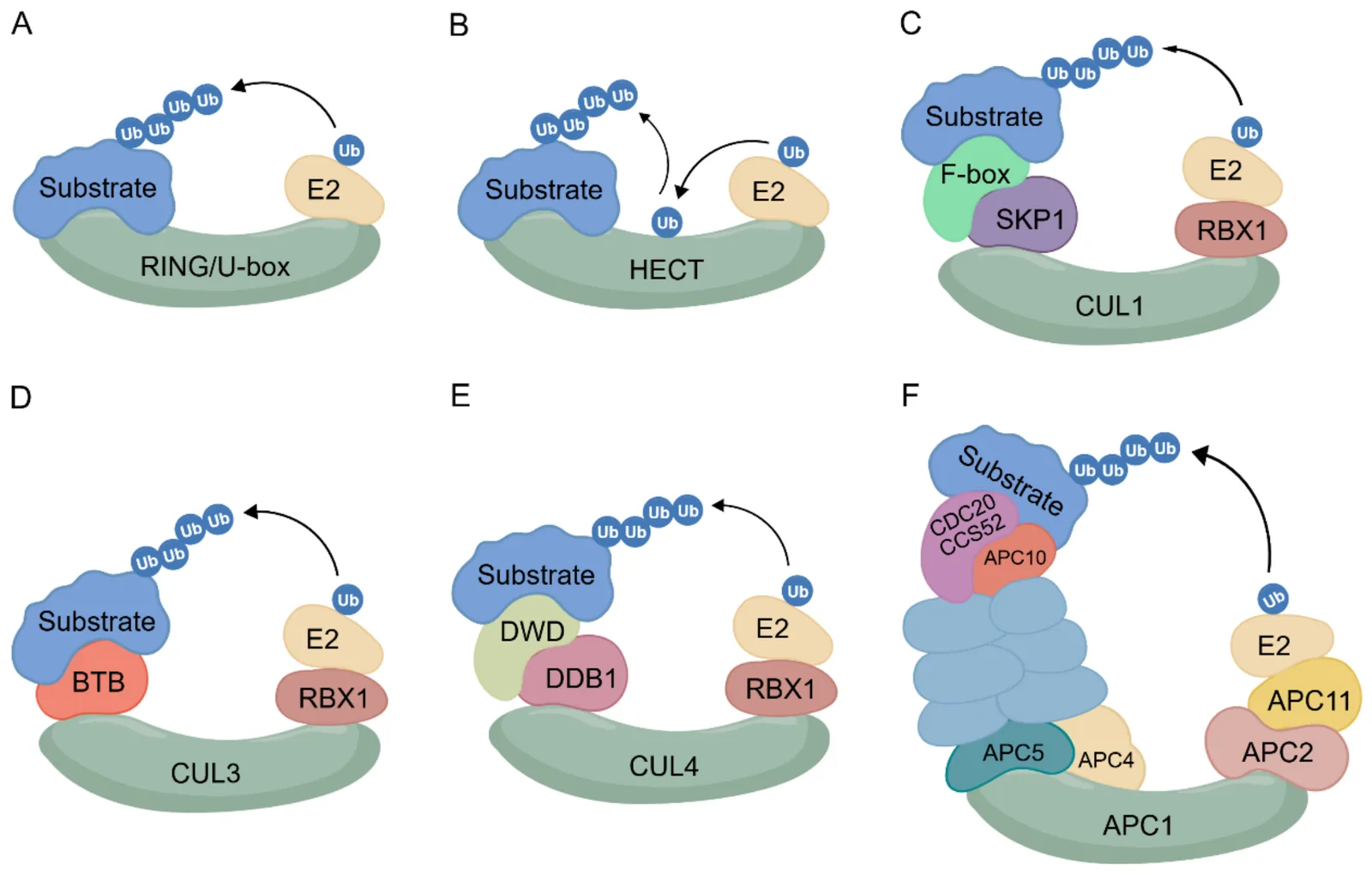
Schematic structures of distinct E3 ubiquitin ligase families. (**A**) Really Interesting New Gene (RING)-type and U-box-type E3 ligases. These ligases are monomeric polypeptides that harbor conserved RING or U-box motifs to bind the E2-ubiquitin intermediate and allosterically facilitate ubiquitin transfer to target substrates. (**B**) Homologous to the E6-AP Carboxyl Terminus (HECT)-type E3 ligases. HECT-type E3 ligases are single polypeptides with a conserved C-terminal HECT domain containing an E2-binding region and a catalytic lobe; the variable N-terminal domain mediates substrate recognition. Distinct from other E3 ligases, HECT-type E3 ligases first catalyze formation of a thioester intermediate between ubiquitin and a cysteine residue within the HECT domain, after which ubiquitin is transferred to lysine residues of substrates. Panels C-F depict different subtypes of Cullin-RING Ligases (CRLs), which are multisubunit E3 complexes composed of a Cullin (CUL) scaffold protein, RING-Box Protein 1 (RBX1, which recruits the E2-ubiquitin intermediate), and a variable substrate recognition module. (**C**) CUL1-type E3 ligase (SKP1-CUL1-F-box, SCF complex). The complex is assembled around a CUL1 scaffold and recruits F-box substrate-recognition proteins via the adaptor S-Phase Kinase-Associated Protein 1 (SKP1). (**D**) CUL3-type E3 ligase (CUL3-BTB/POZ complex). This complex uses CUL3a/CUL3b as the scaffold, and its substrate recognition subunits are Broad-Complex, Tramtrack and Bric-à-Brac/Poxvirus and Zinc Finger (BTB/POZ) domain-containing proteins that directly bind CUL3 and recruit target substrates without additional adaptor proteins. (**E**) CUL4-type E3 ligase (CUL4-DDB1-DCAF complex). The complex takes CUL4 as the scaffold and employs Damage-Binding Protein 1 (DDB1) as an adaptor to recruit diverse DDB1 and CUL4-Associated Factor (DCAF) and DDB1-Binding WD40 (DWD) substrate-recognition proteins. (**F**) Anaphase-promoting complex/cyclosome (APC/C). The APC/C complex consists of at least 11 subunits; APC1, APC4 and APC5 form the central scaffolding platform, APC2 and APC11 function analogously to CULs and RBX1, respectively, and Cell Division Cycle 20 (CDC20), Cell Cycle Switch 52 (CCS52) and APC10 act as substrate adaptors. Figure created with BioGDP.com [8].

Based on protein architecture, catalytic mechanisms and assembly modes, plant E3 ubiquitin ligases are categorized into four major groups: Really Interesting New Gene (RING), U-box, Homologous to the E6-AP Carboxyl Terminus (HECT), and Cullin-RING ligase (CRL) families [5] (Figure 2). CRLs are multisubunit RING-type E3 complexes assembled around a Cullin scaffold. The C-terminal region of Cullin recruits RING-Box Protein (RBX) family RING proteins to capture E2-Ub intermediates, whereas its N-terminal domain associates with adaptor proteins and substrate-recognition subunits. Plant CRL complexes are further classified into four main subtypes according to scaffold isoforms and substrate receptor modules: (1) CRL1 (SKP1-Cullin 1-F-box, SCF complex): Scaffolded by Cullin 1 (CUL1), which interacts with F-box substrate receptors via S-Phase Kinase-Associated Protein 1 (SKP1), which is designated ASK in *Arabidopsis thaliana*. SCF complexes are the best-characterized CRLs in plant hormone signaling, including SCF^COI1^ (jasmonic acid, JA), SCF^TIR1^ (auxin), SCF^SYL1^ (Gibberellin, GA) and SCF^D3^ (strigolactone, SL). (2) CRL3 (Cullin 3-BTB/POZ complex): Assembled on Cullin 3a (CUL3a) and Cullin 3b (CUL3b) scaffolds. Substrate recognition is achieved by Broad-Complex, Tramtrack and Bric-à-Brac/Poxvirus and Zinc Finger (BTB/POZ) domain-containing proteins that directly bind CUL3 and recruit target proteins without additional adaptors. CRL3s extensively participate in plant stress responses and immune regulation. (3) CRL4 (Cullin 4-DDB1-DCAF complex): Centered on Cullin 4 (CUL4), which employs DNA Damage-Binding Protein 1 (DDB1) as an adaptor to recruit diverse substrate receptors, including DDB1 and CUL4-Associated Factor (DCAF) and DDB1-Binding WD40 (DWD) proteins. CRL4s function in photomorphogenesis, DNA damage responses and host–pathogen interactions [9,10]. (4) APC/C (Anaphase-Promoting Complex/Cyclosome): Broadly classified as a multisubunit Cullin-family E3 complex. Its catalytic core comprises the Cullin-related APC2 and RING-H2 APC11 subunits, functionally analogous to the Cullin-RBX catalytic module of canonical CRLs. The APC/C is an enormous complex containing at least 11 core subunits and lacks intrinsic substrate specificity. Target selection depends entirely on two interchangeable coactivators: Cell Division Cycle 20 (CDC20) and Cell Cycle Switch 52 (CCS52, plant orthologs of CDH1). The APC10 (DOC1) subunit cooperates with coactivators to recognize substrates bearing D-box and KEN-box motifs. While canonical CRL complexes predominantly regulate hormone signaling, protein homeostasis and basal immunity, APC/C primarily governs unidirectional progression of the cell cycle [11,12].

E3 ligases catalyze covalent attachment of ubiquitin to lysine residues of target proteins, generating monoubiquitination, multiubiquitination or diverse polyubiquitin chains [13]. Monoubiquitination occurs when the C-terminal glycine of ubiquitin forms an isopeptide bond with the ε-amino group of a substrate lysine. Subsequently, any of the seven lysine residues within ubiquitin (K6, K11, K27, K29, K33, K48 and K63) can serve as acceptor sites for chain elongation, producing polyubiquitin chains with distinct topologies [14]. The biological outcome of ubiquitination is jointly determined by modification type (mono- versus polyubiquitination), chain length (≤4 or >4 ubiquitin moieties) and linkage topology. The well-studied K48-linked polyubiquitin chains mainly mediate proteasomal degradation of substrates; K63-linked chains generally do not trigger degradation but participate in protein trafficking and signaling complex assembly. Monoubiquitination primarily modulates the subcellular localization and chromatin association of target proteins [13].

The reversibility of ubiquitination is controlled by deubiquitinating enzymes (DUBs). Most DUBs are cysteine proteases capable of cleaving ubiquitin isopeptide bonds. DUBs can trim polyubiquitin chains or remove intact ubiquitin moieties from substrates, thereby reversing ubiquitin-dependent signaling, stabilizing target proteins and maintaining cellular ubiquitin homeostasis [15].

The UPS regulates nearly all biological processes in plants, including growth, development, stress responses and immunity [7]. Early studies revealed that Tobacco mosaic virus (TMV) infection upregulates transcripts encoding tobacco E1 ubiquitin-activating enzyme; perturbation of ubiquitin-dependent proteolysis drastically alters tobacco responses to viral challenge, indicating UPS involvement in antiviral defense [16,17]. During long-term plant–virus coevolution, the UPS has become one of the major battlefields [18]. Plants deploy the UPS to eliminate viral components and activate hormone defense cascades, establishing multilayered antiviral barriers. Conversely, viruses have evolved diverse effector proteins to interfere with and hijack core UPS components, suppressing host immunity and remodeling the intracellular microenvironment to support infection and proliferation. Recent years have witnessed accumulating evidence illuminating UPS-mediated plant–virus interactions. This review systematically summarizes the positive roles of the UPS in plant antiviral immunity, dissects molecular strategies adopted by viruses to hijack the UPS for infection, discusses current limitations and future research directions, and provides a theoretical basis for unraveling viral pathogenic mechanisms and developing broad-spectrum antiviral strategies.

## 2. UPS-Mediated Targeted Degradation of Viral Proteins Restricts Viral Infection

### 2.1. Degradation of Viral Suppressors of RNA Silencing (VSR)

**Figure 3:**
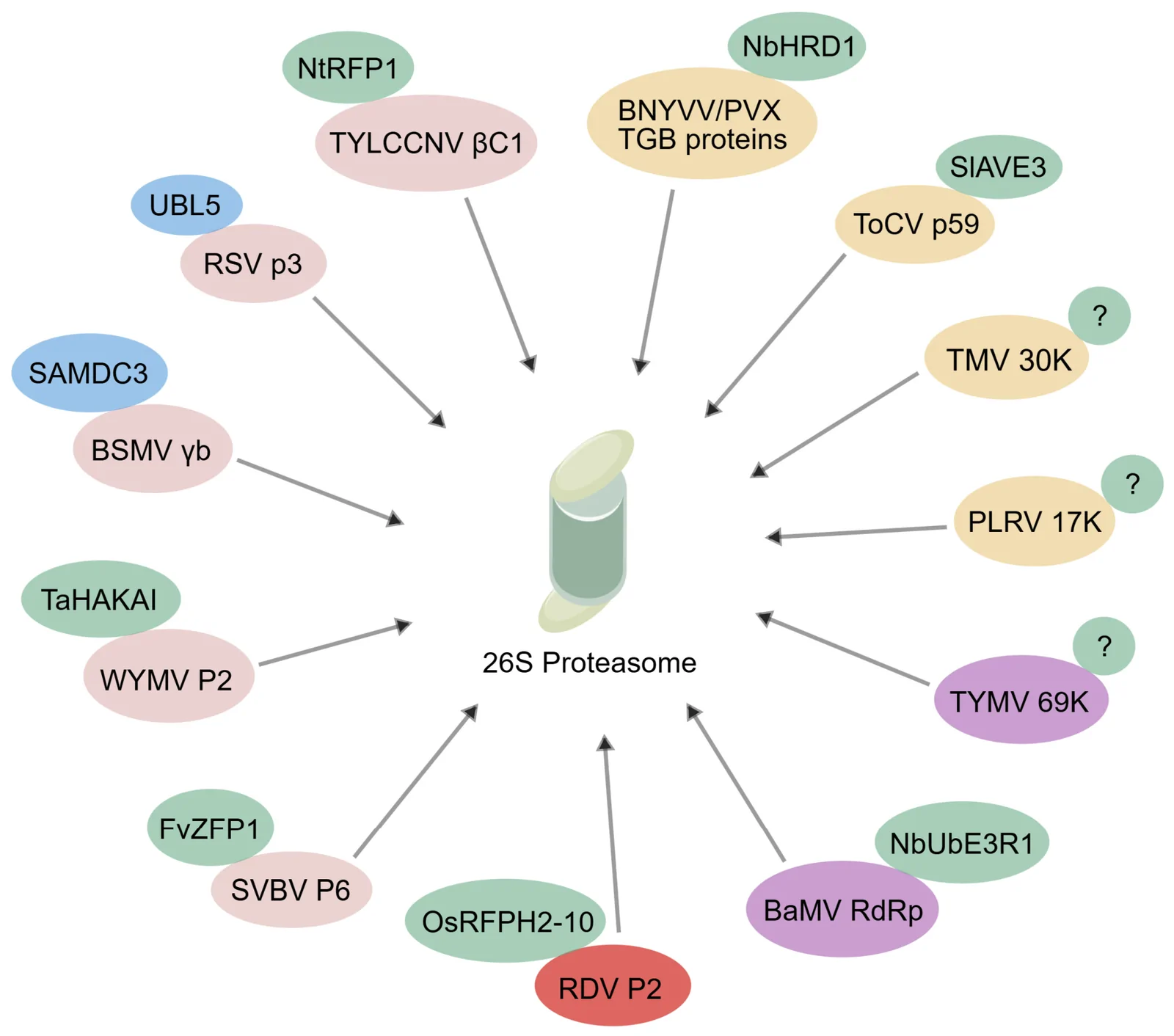
Host proteins target viral proteins for degradation via the ubiquitin-mediated proteasome pathway. Viral proteins are recognized by distinct E3 ubiquitin ligases and then delivered to the 26S proteasome for degradation. Pink ovals denote virus-encoded RNA silencing suppressors, yellow ovals represent virus-encoded Movement Proteins (MPs), purple ovals stand for virus-encoded replicases, red oval indicates virus-encoded Coat Protein (CP), and green ovals denote host E3 ubiquitin ligases. Question marks indicate unidentified E3 ubiquitin ligases, and blue ovals represent other relevant host proteins. Figure created with BioGDP.com [8].

Viruses rely on encoded core proteins to complete replication and cell-to-cell movement. RNA silencing constitutes a central plant immune pathway against viruses. Viruses counteract RNA silencing by expressing VSRs, which represent key viral pathogenic effectors [19,20]. Accumulating data demonstrate that plants employ E3 ubiquitin ligases that specifically recognize VSRs, trigger their ubiquitination and deliver modified VSRs to the 26S proteasome for degradation, thus relieving VSR-mediated inhibition of host RNA silencing (Figure 3).

The βC1 protein encoded by the satellite DNA of Tomato yellow leaf curl China virus (TYLCCNV) acts as a potent VSR. The tobacco Ring Finger Protein 1 (NtRFP1) specifically interacts with βC1 and mediates its K48-linked polyubiquitination and degradation. Silencing *NtRFP1* leads to excessive βC1 accumulation and enhanced susceptibility, whereas overexpression alleviates disease symptoms [21]. The Rice stripe virus (RSV) p3 protein functions as a VSR. Ubiquitin-Like Protein UBL5 from rice and *Nicotiana benthamiana* drives degradation of p3. Silencing *NbUBL5* causes p3 overaccumulation and promotes RSV proliferation, while *NbUBL5* overexpression confers resistance and suppresses RSV infection [22]. Recent work identified wheat TaHAKAI, a protein with both methyltransferase and E3 ubiquitin ligase activity, which targets the Wheat yellow mosaic virus (WYMV) VSR P2 and triggers its degradation to block viral infection [23].

The Strawberry vein banding virus (SVBV) P6 protein serves as a symptom determinant, VSR and transcriptional activator. Yang et al. demonstrated that strawberry Zinc Finger Protein 1 (FvZFP1) is an antiviral protein functioning as both an E3 ligase and transcription factor. Via its ubiquitin ligase activity, FvZFP1 recognizes SVBV P6 and mediates its ubiquitination and degradation to inhibit infection; meanwhile, FvZFP1 directly binds the viral promoter to suppress viral transcription [24]. S-Adenosylmethionine Decarboxylase 3 (SAMDC3) from wheat and *N. benthamiana* interacts with Barley stripe mosaic virus (BSMV) γb protein and acts as a molecular bridge to recruit γb into the UPS degradation pathway. Silencing *SAMDC3* markedly elevates viral accumulation and exacerbates symptoms, whereas *SAMDC3* overexpression enhances γb ubiquitination, accelerates γb turnover and boosts plant resistance [25].

### 2.2. Degradation of Viral Movement Proteins (MPs)

Following single-cell infection, plant viruses spread between cells through plasmodesmata, a process dependent on virally encoded MPs. MPs bind viral nucleic acids and modulate plasmodesmal permeability to facilitate intercellular viral trafficking [26,27]. Host-mediated UPS-dependent degradation of MPs represents an efficient antiviral route (Figure 3). The Tomato chlorosis virus (ToCV) p59 protein participates in virion assembly and promotes cell-to-cell movement. Tomato Antiviral E3 Ubiquitin Ligase (SlAVE3) ubiquitinates p59 and targets it for degradation to restrict systemic ToCV infection [28]. Proteasome inhibitor treatment results in massive accumulation of TMV 30K MP at the perinuclear Endoplasmic Reticulum (ER) and enhanced MP stability, implying that UPS-mediated turnover of this MP limits systemic viral spread and alleviates ER structural damage caused by MP overaccumulation [29]. The Turnip yellow mosaic virus (TYMV) 69K MP exhibits in vitro instability, undergoes polyubiquitination and serves as a substrate of the 26S proteasome [30]. The Potato leafroll virus (PLRV) 17K MP aggregates extensively upon proteasome inhibition [31].

The ER-associated degradation (ERAD) pathway utilizes the UPS to eliminate aberrant ER-resident proteins and maintain cellular proteostasis. Emerging evidence indicates that ERAD contributes to plant antiviral immunity by regulating the stability of viral MPs. Ju et al. reported that turnover of Potato virus X (PVX) TGBp3 is controlled by ERAD, and proteasome inhibition delays its degradation [32]. Recently, Guo et al. found that *N. benthamiana* HMG-CoA Reductase Degradation Protein 1 (NbHRD1) possesses functional E3 ubiquitin ligase activity. NbHRD1 binds TGB MPs of Beet necrotic yellow vein virus (BNYVV) and PVX, induces their ubiquitination and subsequent UPS-dependent degradation, thereby obstructing cell-to-cell viral movement and suppressing infection [33].

### 2.3. Degradation of Viral Replicase

Viral RNA-dependent RNA polymerase (RdRp) serves as the core catalytic protein for genomic replication of positive-sense RNA viruses. It synthesizes progeny viral RNAs using viral RNA as the template and directly determines viral replication efficiency [34]. Accordingly, RdRp constitutes a crucial target for host defense, and the UPS represses viral replication by mediating the degradation of viral replicase (Figure 3). Camborde et al. reported that the 66K RdRp encoded by TYMV is degraded by the host UPS at the late infection stage, which attenuates viral infectivity [35]. The RING-domain-containing ubiquitin E3 ligase NbUbE3R1 from *N. benthamiana* binds to the replicase of Bamboo mosaic virus (BaMV) to restrict viral replication. Knock-down of *NbUbE3R1* via Tobacco rattle virus (TRV)-mediated Virus-Induced Gene Silencing (VIGS) markedly elevates BaMV accumulation, whereas overexpression of *NbUbE3R1* and its truncated mutants inhibits viral proliferation [36].

### 2.4. Degradation of Viral Coat Protein (CP)

CP is a multifunctional structural protein of viruses. Apart from encapsidating and protecting viral genomes, CP participates in viral assembly, cell-to-cell and long-distance movement, as well as vector-mediated transmission [37]. Under certain circumstances, plant UPS targets CP and interferes with viral infection by modulating CP abundance (Figure 3). The rice RING-H2-type E3 ubiquitin ligase OsRFPH2-10 interacts with the P2 protein of Rice dwarf virus (RDV) and mediates UPS-dependent degradation of P2. Overexpression of *OsRFPH2-10* at the early infection stage significantly reduces viral accumulation [38].

## 3. Viruses Hijack the UPS to Promote Infection

During the long-term evolutionary arms race, viruses have evolved diverse molecular strategies to target and hijack the host UPS. By disrupting the assembly of ubiquitin complexes, mediating the targeted degradation of host disease-resistant proteins and other approaches, viruses suppress plant immune responses and remodel the intracellular microenvironment to support their own replication and spread. The documented modes of viral UPS-mediated counter-defense, corresponding effector proteins and action targets reported to date are summarized in Table 1.

### 3.1. Viral Proteins Disrupt Functions of Core UPS Complexes

#### 3.1.1. Interaction with SKP1 Impairs SCF Complex Assembly

The βC1 protein encoded by Cotton leaf curl Multan virus (CLCuMuV) satellite DNA interacts with the SCF core subunit NbSKP1, preventing assembly of NbSKP1 with NbCUL1 and disrupting the activity of SCF^COI1^ and SCF^SYL1^ ubiquitin complexes, consequently facilitating CLCuMuV infection and symptom development [39]. Auxin/Indole-3-Acetic Acid (Aux/IAA) proteins are short-lived nuclear repressors of auxin signaling. SCF^TIR1^-mediated ubiquitination and degradation of Aux/IAA proteins are essential for auxin signal activation. The ToCV-encoded p22 protein binds the C-terminal domain of NbSKP1.1, destabilizing SCF^TIR1^ complexes, blocking auxin signaling and enhancing viral susceptibility [40].

#### 3.1.2. Targeting the COP9 Signalosome (CSN)

Related to Ubiquitin (RUB; known as Nedd8 in fungi and animals) is a ubiquitin-like modifier in plants. Covalent RUB modification of Cullin subunits is required for SCF complex activation. The CSN harbors deRUBylation activity that removes RUB moieties from Cullins, a process essential for cyclic activation and sustained function of SCF E3 ligases [61,62].

C2/L2 proteins encoded by Tomato yellow leaf curl Sardinia virus (TYLCSV), Tomato yellow leaf curl virus (TYLCV) and Beet curly top virus (BCTV) interact with CSN5 of the COP9 signalosome, interfering with CUL1 deRUBylation and broadly suppressing SCF activity. This leads to widespread perturbation of SCF-controlled hormone and developmental pathways, among which jasmonate signaling is most severely disrupted by C2. Exogenous methyl jasmonate (MeJA) application restricts viral infection, verifying that C2 promotes infection via suppression of jasmonate-mediated defenses [41]. The Rice black-streaked dwarf virus (RBSDV) P5-1 protein binds rice OsCSN5A, disrupts CSN-mediated OsCUL1 deRUBylation, impairs SCF activity, inhibits jasmonate signaling and enhances RBSDV infectivity. Notably, unlike C2-overexpressing Arabidopsis, exogenous jasmonate fails to restore antiviral resistance in P5-1 transgenic rice [42].

### 3.2. Viruses Manipulate the UPS to Antagonize Key Host Defense Regulators

#### 3.2.1. Interference with RNA-Directed DNA Methylation (RdDM)-Mediated Transcriptional Gene Silencing (TGS)

Cytosine DNA methylation represents a reversible epigenetic modification regulating gene expression, transposon silencing and transgene silencing. Genetic and biochemical analyses have identified dozens of components functioning in the RdDM pathway, including specific subunits of RNA polymerase IV (Pol IV) and RNA polymerase V (Pol V). Current functional models categorize these proteins into three core modules: Pol IV-dependent small interfering RNA (siRNA) biogenesis, Pol V-mediated de novo DNA methylation, and chromatin remodeling processes governed by histone modification, nucleosome positioning and higher-order chromatin organization. The key core factors within this pathway include the methyl donor S-Adenosylmethionine (SAM), Domains-Rearranged Methyltransferase 2 (DRM2), Methyltransferase 1 (MET1), chromomethyltransferases CMT2 and CMT3, as well as RNA-Directed DNA Methylation 1 (RDM1) [63,64]. Against DNA viruses such as geminiviruses, plants deploy DNA methylation-dependent TGS to inhibit viral genome transcription, constituting a critical epigenetic defense [65]. To evade TGS, multiple DNA viruses encode effectors that hijack the host UPS to degrade components of DNA methylation pathways and attenuate epigenetic silencing (Table 1).

Upon Beet severe curly top virus (BSCTV) infection of Arabidopsis rosette leaves, the viral replication initiator protein (Rep) induces expression of *Variant in Methylation 5* (*VIM5*), an E3 ubiquitin ligase that directly targets DNA methyltransferases MET1 and CMT3 for UPS-dependent degradation. This reduces symmetric methylation within the viral C2-C3 promoter, relieves transcriptional repression of viral C2 and C3 genes, and triggers symptomatic infection [43]. Beyond direct degradation of methyltransferases, viruses remodel host methylation metabolism via the UPS. BSCTV C2 interacts with S-Adenosylmethionine Decarboxylase 1 (SAMDC1), a key enzyme maintaining SAM/decarboxylated SAM homeostasis for transmethylation reactions. C2 hijacks the 26S proteasome pathway to stabilize SAMDC1 in a non-proteolytic manner. Excessive SAMDC1 depletes the methyl donor SAM, causing global hypomethylation of host DNA and counteracting virus-targeted epigenetic silencing [44].

The Rice grassy stunt virus (RGSV) P3 protein binds the *Somatic Embryogenesis Receptor Kinase 4* (*SERK4*) promoter and upregulates *SERK4* transcription, disrupting the balance between plant growth and immunity. SERK4 directly phosphorylates E3 ligase P3-Inducible Protein 1 (P3IP1) and activates its catalytic activity. Activated P3IP1 recognizes and mediates ubiquitination and degradation of NRPD1a (Pol IV subunit), a core RdDM component. This suppresses Pol IV-dependent antiviral RdDM and weakens epigenetic resistance against RGSV [45,46].

#### 3.2.2. Ubiquitination-Mediated Degradation of Post-Transcriptional Gene Silencing (PTGS) Core Factors

RNA interference (RNAi), also termed RNA silencing, is an evolutionarily conserved sequence-specific gene regulatory mechanism and the primary innate plant defense against viruses. Viral double-stranded RNAs are processed by Dicer-Like (DCL) enzymes into virus-derived small interfering RNAs (vsiRNAs), which are loaded into Argonaute (AGO) proteins to assemble RNA-Induced Silencing Complex (RISC) that inhibits viral proliferation at the post-transcriptional level. The RNA-Dependent RNA Polymerase 6 (RDR6)-Suppressor of Gene Silencing 3 (SGS3) complex synthesizes secondary siRNAs to amplify defense signals [66]. AGO1 functions as a master effector of PTGS against RNA viruses [66]. As a counter-defense strategy, diverse plant viruses utilize the UPS to trigger degradation of silencing factors including AGO1 and SGS3, blocking RNAi immunity to facilitate infection (Table 1).

The PVX VSR P25 interacts with AGO1 and drives its 26S proteasome-dependent degradation, suppressing antiviral RNA silencing [47]. Areca palm velarivirus 1 (APV1) p26 is a multifunctional VSR whose host target is SGS3; p26 overexpression accelerates ubiquitination-dependent SGS3 turnover to inhibit host immunity [48]. Double-Stranded RNA (dsRNA)-Binding Protein 7.1 (DRB7.1) and DRB7.2 harbor a single dsRNA-binding domain and serve as essential auxiliary factors maintaining DCL4 antiviral activity in the absence of DRB4. They share no sequence homology with DRB4 and provide functional redundancy via structural substitution. The Turnip crinkle virus (TCV) CP directly binds DRB7.1/7.2 and mediates their UPS-dependent degradation, disabling this backup immune pathway [49].

#### 3.2.3. Manipulation of Central Components of Diverse Hormone Pathways

Plant hormones are core signaling molecules coordinating plant growth and responses to biotic and abiotic stresses [67,68]. Complex hormonal crosstalk precisely modulates antiviral immunity [69]. UPS-driven ubiquitination and degradation of target proteins constitute a foundational molecular switch governing hormone signal activation and transduction [70,71]. During coevolution, plant viruses have evolved diverse pathogenic strategies: viral effectors target UPS components and hijack ubiquitin-dependent turnover of central hormonal regulators, disrupting salicylic acid (SA)-, GA-, auxin- and SL-mediated antiviral networks to establish a permissive intracellular environment for viral propagation (Table 1).

Nonexpressor of Pathogenesis-Related Genes 1 (OsNPR1), a master regulator of SA signaling, simultaneously activates jasmonate cascades. OsNPR1 dissociates the Jasmonate ZIM-Domain Protein (OsJAZ)-Myelocytomatosis Transcription Factor 2 (OsMYC2) complex and enhances the transcriptional activity of OsMYC2, thereby triggering JA signaling and synergistically regulating rice antiviral immunity. RSV P2, Southern rice black-streaked dwarf virus (SRBSDV) SP8 and Rice stripe mosaic virus (RSMV) M proteins disrupt physical interactions between OsNPR1 and OsMYC2/OsMYC3, interfering with OsNPR1-mediated SA-JA crosstalk to promote viral virulence. Furthermore, RSV P2 strengthens OsNPR1-OsCUL3a association to accelerate OsNPR1 degradation and suppress SA-mediated resistance [50]. Li et al. demonstrated that phylogenetically divergent viral proteins physically promote binding between gibberellin receptor Gibberellin Insensitive Dwarf1 (OsGID1) and Slender Rice 1 (SLR1), specifically inducing SLR1 degradation and attenuating SLR1-dependent broad-spectrum antiviral immunity [51]. RDV CP P2 interacts with auxin signaling Aux/IAA protein OsIAA10, blocking OsIAA10-Transport Inhibitor Response 1 (OsTIR1) interaction and inhibiting 26S proteasome-dependent OsIAA10 turnover, consequently suppressing auxin signaling and enhancing viral pathogenicity. OsIAA10 knockout improves rice resistance to RDV, whereas transgenic rice overexpressing wild-type OsIAA10 or stable dominant-negative OsIAA10 recapitulates RDV-infected phenotypes and exhibits heightened susceptibility to RDV [52].

A recent Cell publication confirmed that SL signaling positively regulates rice antiviral RNA silencing. Activated SL signaling stabilizes Rice Pathogen-Responsive NAC Transcription Factor 131 (ONAC131), which cooperates with MYB Important for Drought Response 1 (MID1) to induce transcription of antiviral core genes *RDR1* and *RDR6*, reinforcing RNAi-mediated defense. RGSV P3 competitively binds DWARF14 (D14) and occupies the D14-DWARF3 (D3) interaction interface, blocking SL signal transduction. This inhibition impairs activation of *RDR1*/*RDR6* and weakens RNAi-based disease resistance [53].

### 3.3. Viral Modification of Viral Protein Ubiquitination Enables Evasion of Host Degradation

The host UPS targets essential viral proteins to restrict infection. Several viruses have evolved to encode intrinsic DUBs to counteract host-mediated viral protein turnover. The TYMV 66K RdRp is recognized and degraded by the plant UPS at late infection stages, limiting viral replication [35]. The TYMV 98K replication protein contains an Ovarian Tumor (OTU)-type cysteine protease (PRO) domain with DUB activity, which specifically removes ubiquitin moieties from 66K RdRp, prevents its proteasomal degradation and promotes infection [54]. These findings reveal an intricate regulatory network interconnecting ubiquitination, deubiquitination and viral protein homeostasis to modulate RdRp abundance and viral infectivity.

Distinct from TYMV encoding viral DUBs, potyviruses and numerous other plant viruses adopt an alternative strategy: hijacking host DUBs. Soybean mosaic virus (SMV) Cylindrical Inclusion (CI) protein interacts with and stabilizes host ubiquitin-specific protease UBP14 by preventing UBP14 ubiquitination. CI recruits UBP14 to viral replication complexes, where UBP14 trims ubiquitin chains from viral replication proteins to avoid proteasomal clearance. Beyond potyviruses, PVX TGBp1 and rhabdovirus P protein stabilize UBP14 via suppression of its ubiquitination. This indicates convergent evolution across diverse viral lineages to deploy effectors targeting UBP14 and exploit host deubiquitination to escape antiviral protein degradation [55].

### 3.4. Viral Remodeling of Hormone Pathways via the UPS Facilitates Vector Transmission

Most plant viruses rely on insect vectors for spread, and vector insects often exhibit improved survival and reproduction on virus-infected plants [72]. JA represents the most extensively investigated hormone in virus-modulated plant-insect interactions, as JA directly mediates plant defense against herbivorous insects and regulates biosynthesis of volatile blends [73]. Li et al. showed that TYLCV C2 suppresses plant defense by interacting with the N-terminal ubiquitin domain of Ubiquitin-40S Ribosomal Protein S27a (RPS27A). This interaction inhibits ubiquitination and 26S proteasome-mediated degradation of JAZ1, repressing jasmonate-dependent defense responses and MYC2-controlled terpene synthase expression. Reduced terpene emission renders infected plants more attractive to vector insects, facilitating viral spread in fields and establishing a synergistic virus-vector pathogenic cycle. Papaya leaf curl China virus (PaLCuCNV) employs an identical infection strategy [56]. Cucumber mosaic virus (CMV) 2b directly binds JAZ proteins and suppresses JA-triggered JAZ degradation, attenuating jasmonate signaling and increasing aphid preference for CMV-infected plants [57]. The pathogenic βC1 protein encoded by Tomato yellow leaf curl China betasatellite (TYLCCNB) associated with TYLCCNV interacts with tobacco NtSKP1, inhibits JAZ1 turnover, dampens jasmonate defenses and enhances whitefly fitness on TYLCCNV/TYLCCNB-infected plants [58].

### 3.5. Viral UPS Hijacking Remodels the Cell Cycle to Establish Replication Niches

The BSCTV symptom determinant C4 induces aberrant cell division in Arabidopsis. C4 upregulates expression of the RING-type E3 ligase *Kip-Related Protein* (*RKP*), which interacts with Inhibitor of Cyclin-Dependent Kinase/Kip-Related Protein (ICK/KRP) cell cycle repressors. BSCTV infection reduces ICK2/KRP2 abundance. *RKP* mutation or *ICK1/KRP1* overexpression decreases plant susceptibility to BSCTV. Therefore, C4 remodels host–cell cycle progression by modulating ICK/KRP levels to promote BSCTV infection [59].

The APC/C is an evolutionarily conserved E3 ligase complex driving ordered cell cycle progression via polyubiquitination and degradation of target proteins. Under physiological conditions, tomato SlCDC20.1/3 associate with Retinoblastoma-Related 1 (SlRBR1) and mediate degradation of D-type cyclin SlCycD1;1, stabilizing inhibitory complexes of SlRBR1 and Early-Region-2 Transcription Factor (SlE2F), and maintaining cell cycle quiescence. Viral proteins encoded by the DNA geminivirus TYLCV and RNA crinivirus ToCV both directly target tomato SlCDC20.1 and SlCDC20.3. Viruses manipulate APC/C^SlCDC20^ function through two divergent mechanisms: TYLCV Rep directly activates APC/C^SlCDC20^ to trigger SlRBR1 degradation, whereas TYLCV C2 and ToCV p27 inhibit APC/C^SlCDC20^ activity, blocking SlCycD1;1 degradation and indirectly promoting SlCycD1;1-dependent SlRBR1 depletion to release free SlE2F transcription factors. Liberated SlE2F activates transcription of DNA polymerase genes (*SlPols*) and heat shock protein 70 gene (*SlHSP70*), supplying host factors essential for replication of DNA and RNA viruses, respectively. Aberrant dissociation of RBR1-E2F complexes also causes tomato growth retardation. These observations demonstrate that phylogenetically distinct viruses hijack core UPS machinery to reprogram the cell cycle and generate specialized nuclear microenvironments supporting viral replication [60].

## 4. Conclusions and Perspectives

### 4.1. Conclusions

As a central regulatory hub governing plant proteostasis and signal transduction, the UPS has evolved into a strategic battlefield for host–virus conflicts over evolutionary timescales. This review systematically elaborates the bidirectional regulatory logic underlying UPS-dependent plant–virus interactions. On one hand, plants exploit the substrate selectivity of E3 ubiquitin ligases to precisely mark viral VSRs, MPs and other functional factors for proteasomal clearance, forming a vital innate immune barrier against infection. On the other hand, viruses have evolved multilayered counter-defensive strategies under evolutionary pressure. Viral effectors interfere with E3 complex assembly and rewire intracellular signaling networks, disrupting host defense homeostasis and generating intracellular microenvironments permissive for infection and even vector transmission. Consequently, the UPS is converted from a plant defensive weapon into a powerful tool facilitating viral pathogenicity.

### 4.2. Perspectives

Extensive progress has been achieved in deciphering molecular mechanisms linking the UPS to plant–virus interactions [18]; nevertheless, numerous critical questions remain unresolved. Current research predominantly focuses on K48-linked polyubiquitination driving proteasomal degradation in antiviral immunity. The regulatory roles of monoubiquitination and non-degradative K63-linked polyubiquitin chains in viral intracellular trafficking and immune signaling complex assembly lack systematic characterization. Regarding E3 ligases, the plant–virus arms race mediated by monomeric RING/U-box ligases and SCF complexes has been relatively well explored, whereas functions of large multisubunit E3 complexes including CRL3, CRL4 and APC/C in antiviral defense remain largely unstudied. Whether viruses target and hijack these mega-complexes to remodel cellular environments, together with their downstream signaling cascades, requires further experimental validation.

Most existing experiments employ binary infection models with single viral inoculation. However, field-grown crops commonly sustain mixed viral infections. It remains unclear whether competitive interference of diverse viral effectors with UPS pathways alters established hijacking strategies and reshapes overall plant resistance phenotypes. In addition, many mechanistic studies are performed using model plants such as Arabidopsis and *N. benthamiana*, and conclusions lack sufficient genetic and field validation in staple and cash crops. Translation of fundamental knowledge toward resistance breeding is significantly delayed. Future research should prioritize functional complementation and field resistance evaluation of candidate genes in target crops.

The UPS orchestrates core physiological events throughout the plant lifespan, including cell cycle progression, organ morphogenesis and hormone signaling networks [7]. Traditional strategies relying on gene knockout or constitutive overexpression for resistance improvement frequently trigger developmental defects, fertility reduction and yield penalties. Therefore, future translational research should prioritize identification of specific antiviral targets. Two directions merit attention: screening E3 ubiquitin ligases that selectively mediate degradation of viral pathogenic proteins without disrupting normal host growth and metabolism; identifying host interactors of viral effectors and plant susceptibility genes exploited for viral infection. Gene-editing technologies can be utilized to introduce site-specific mutations at critical amino acids mediating protein interactions or moderately attenuate susceptibility gene function. Such modifications block viral hijacking of the UPS at the molecular level, enabling creation of germplasm with durable antiviral resistance while preserving desirable agronomic traits. High-impact research has validated this strategy: single-base editing of the SL receptor D14 (D102N mutation) disrupts interaction between RGSV P3 and D14. This mutation enables rice to evade viral suppression of SL-dependent RNA silencing immunity without impairing native hormone perception, stably conferring resistance to RGSV [53]. This precise targeted modification paradigm offers a reference for developing green control strategies against plant viral diseases via manipulation of UPS pathways.

## Figures and Tables

**Table 1:** Summary of viral proteins that manipulate the plant ubiquitin–proteasome system during infection.

Virus	Virus Protein	Cellular Target	Proposed Mechanism	Proposed Function	Reference
CLCuMuV	βC1	NbSKP1	Binds NbSKP1 and disrupts assembly of SCF^COI1^/SCF^SYL1^ complexes	Suppresses JA/GA-mediated antiviral defense	[39]
ToCV	p22	NbSKP1.1	Binds the C-terminal domain of NbSKP1.1 and destabilizes SCF^TIR1^ complex	Blocks auxin signaling	[40]
TYLCSV/TYLCV	C2	CSN5	Binds CSN5 and interferes with CUL1 deneddylation	Inhibits SCF-dependent hormonal defense pathways	[41]
BCTV	L2				
RBSDV	P5-1	OsCSN5A	Binds OsCSN5A and inhibits OsCUL1 deneddylation	Suppresses JA-mediated antiviral defense	[42]
BSCTV	Rep	MET1 and CMT3	Induces E3 ligase VIM5 to mediate ubiquitination and degradation of MET1/CMT3	Antagonizes DNA methylation silencing of viral genome	[43]
BSCTV	C2	SAMDC1	Hijacks the UPS pathway to stabilize SAMDC1 in a non-degradative manner	Antagonizes DNA methylation silencing of viral genome	[44]
RGSV	P3	OsNRPD1a	Activates E3 ubiquitin ligase P3IP1 to degrade OsNRPD1a	Inhibits the RdDM pathway	[45,46]
PVX	P25	AGO1	Mediates AGO1 degradation via the UPS pathway	Suppresses RNA silencing-based antiviral defense	[47]
APV1	p26	SGS3	Promotes SGS3 degradation via the UPS pathway	Suppresses RNA silencing-based antiviral defense	[48]
TCV	CP	DRB7.1/7.2	Mediates DRB7.1/7.2 degradation via the UPS pathway	Suppresses RNA silencing-based antiviral defense	[49]
RSV	P2	OsNPR1	Enhances OsNPR1-OsCUL3a interaction to promote OsNPR1 degradation	Disrupts SA-JA synergistic antiviral immunity	[50]
RSV SRBSDV	P2 SP8	SLR1	Promotes OsGID1-SLR1 association to induce SLR1 degradation	Attenuates JA-dominated antiviral immunity	[51]
RDV	P2	OsIAA10	Blocks OsIAA10-OsTIR1 interaction and inhibits OsIAA10 degradation	Blocks auxin signaling	[52]
RGSV	P3	D14	Competitively binds D14 and blocks D14-D3 interaction	Blocks strigolactone signaling	[53]
TYMV	98K	66K	Possesses deubiquitinase activity and removes ubiquitin from 66K RdRp	Stabilizes viral replication proteins	[35,54]
SMV	CI	UBP14	Stabilizes UBP14 to deubiquitinate viral proteins	Stabilizes viral replication proteins	[55]
PVX	TGBp1				
SNYV	P				
TYLCV/PaLCuCNV	C2	RPS27A	Binds the ubiquitin domain of RPS27A and inhibits JAZ1 degradation	Suppresses JA pathway and promotes vector transmission	[56]
CMV	2b	JAZ	Directly binds JAZ and inhibits its ubiquitination and degradation	Suppresses JA pathway and promotes vector transmission	[57]
TYLCCNV	βC1	NtSKP1	Binds NtSKP1 and inhibits SCF^COI1^-mediated JAZ1 degradation	Suppresses JA pathway and promotes vector transmission	[58]
BSCTV	C4	ICK/KRP	Induces E3 ligase RKP to mediate ubiquitination and degradation of ICK/KRP	Regulates cell cycle	[59]
TYLCV	Rep	SlCDC20.1/3	Directly activates APC/C^SlCDC20^ complex to mediate SlRBR1 degradation	Regulates cell cycle	[60]
ToCV	p27	SlCDC20.1/3	Inhibits APC/C^SlCDC20^ activity and indirectly promotes RBR1 depletion	Regulates cell cycle	[60]

The full names of all viruses in this table are provided in the Abbreviations Section at the end of the article.

## Data Availability

No new data were created or analyzed in this study. Data sharing is not applicable to this article.

## Abbreviations

CRL	cullin-RING ligase
SKP1	s-phase kinase-associated protein 1
BTB/POZ	broad-complex, tramtrack and bric-à-brac/poxvirus and zinc finger
DDB1	DNA damage-binding protein 1
DCAF	DDB1 and CUL4-associated factor
DWD	DDB1-binding WD40
APC/C	anaphase-promoting complex/cyclosome
CDC20	cell division cycle 20
CCS52	cell cycle switch 52
CSN	constitutive photomorphogenesis 9 signalosome (COP9 signalosome)
RUB	related to ubiquitin
Aux/IAA	auxin/indole-3-acetic acid
DsRNA	double-stranded RNA
DRB	double-stranded RNA-binding protein
SGS3	suppressor of gene silencing 3
RDR	RNA-dependent RNA polymerase
RISC	RNA-induced silencing complex
siRNA	small interfering RNA
TGS	transcriptional gene silencing
PTGS	post-transcriptional gene silencing
RdDM	RNA-directed DNA methylation
VSR	viral suppressor of RNA silencing
ERAD	endoplasmic reticulum-associated degradation
UPS	ubiquitin–proteasome system
ICK/KRP	inhibitor of cyclin-dependent kinase/Kip-related protein
APV1	Areca palm velarivirus 1
BaMV	Bamboo mosaic virus
BCTV	Beet curly top virus
BNYVV	Beet necrotic yellow vein virus
BSCTV	Beet severe curly top virus
BSMV	Barley stripe mosaic virus
CLCuMuV	Cotton leaf curl Multan virus
PaLCuCNV	Papaya leaf curl China virus
PLRV	Potato leafroll virus
PVX	Potato virus X
RDV	Rice dwarf virus
RBSDV	Rice black-streaked dwarf virus
RGSV	Rice grassy stunt virus
RSMV	Rice stripe mosaic virus
RSV	Rice stripe virus
SMV	Soybean mosaic virus
SRBSDV	Southern rice black-streaked dwarf virus
SVBV	Strawberry vein banding virus
TCV	Turnip crinkle virus
TMV	Tobacco mosaic virus
ToCV	Tomato chlorosis virus
TRV	Tobacco rattle virus
TYLCV	Tomato yellow leaf curl virus
TYLCCNV	Tomato yellow leaf curl China virus
TYLCCNB	Tomato yellow leaf curl China betasatellite
TYLCSV	Tomato yellow leaf curl Sardinia virus
TYMV	Turnip yellow mosaic virus
WYMV	Wheat yellow mosaic virus
